# Evaluation of noise hazard during the holmium laser enucleation of prostate

**DOI:** 10.1186/s12894-017-0246-y

**Published:** 2017-08-31

**Authors:** Huan Xu, Yan-bo Chen, Meng Gu, Qi Chen, Zhong Wang

**Affiliations:** Department of Urology, Shanghai 9th People’s Hospital, Shanghai Jiaotong University School of Medicine, 639 Zhi Zaoju Road, Shanghai, 200011 China

## Abstract

**Background:**

To evaluate noise hazard during holmium laser enucleation of the prostate (HoLEP), we designed a study to detect such a risk in this procedure.

**Methods:**

This study was conducted over a 12-month period on 223 patients with benign prostatic hyperplasia (BPH), 121 of whom underwent HoLEP while those remaining underwent transurethral resection of the prostate (TURP). A sound level meter was used to detect the exposure of surgeons to noise. The recordings used were in accordance with the standards set by the Occupational Safety and Health Administration (OSHA) and the United States Environmental Protection Agency. Moreover, each of the 43 surgeons participating in a BPH discussion conference answered the questionnaire on the influence of noise, and 33 surgeons in our department volunteered for blood pressure monitoring post-surgically.

**Results:**

The sound level produced by a high-powered holmium laser emitter during HoLEP was 67.37 ± 0.13 dB, which was significantly higher than the sound heard during TURP (46.41 ± 0.29 dB, *P* < 0.01). The 65–70 dB noise during HoLEP was proved to be a safe level in accordance with the OSHA standards. However, this level was considerably greater than the stated 55 dB. Moreover, it exceeded the normal communication protective level of 60 dB. In the analysis of responses from the surgeons, the HoLEP group obtained an average score that reflected disturbance caused by the laser emitter and an increase in average systolic pressure relative to that in the TURP group.

**Conclusions:**

The noise level during HoLEP is within hearing conservation levels. However, the noise disturbs intrateam communication and concentration during surgery. Some surgeons may experience discomfort post-surgically, but no significant difference among the groups is indicated. The findings suggest that measures should be taken to address the noise caused by the laser emitter during HoLEP.

## Background

With the development of minimally invasive surgery, holmium laser enucleation of prostate (HoLEP) has been widely used worldwide and is considered as the “new golden standard” for benign prostate hyperplasia (BPH) [1]. A large number of studies have focused on the effects of surgery on patients, but few have evaluated the effects of surgery on surgeons. One of the potential risks of surgery for doctors is noise exposure. To reduce the negative effects of noise hazard, national and European community directives as well as United Nations guidelines recommend a 55 db threshold for work requiring concentration such as “decisions under time pressure,” “decisions with severe consequences,” or “examinations and operations by physicians, meetings, research, teaching.” [2]. Ambient noise exhibits a tendency to affect performance during surgery, causing decreased concentration and mental loading during surgery; dexterity is also decreased, as shown in the simulation video [3, 4]. Noise volume is associated with surgical site infection, which may cause serious post-surgical complications [5]. In many medical areas, such as orthopedic and dental department, noise hazards have recently been reported and is given considerable attention [6, 7]. The present study is the first to evaluate the influence of noise produced during HoLEP on urological surgeons.

## Methods

This study was performed over a period of 12 months on 223 patients. Among the patients, 121 underwent HoLEP using a high-powered holmium laser with a 100 W continuous flow and with power settings of 80–100 W at 2–1.5 J/s and 50–40 Hz. The remaining 102 patients underwent TURP. Average sound levels were recorded during surgery, and the sound range was measured during the procedure. The location chosen was 40 cm away from the surgeons’ head. The sound level meter (Control Company, Friendswood, TX) produced by Thomas Scientific was used to measure the sound in decibels.

In addition, each of the 59 surgeons participating in a BPH operating conference responded to questionnaire regarding the effect of HoLEP noise on the performance of the surgeon, most of whom were skilled in both HoLEP and TURP. Excluding the incomplete questionnaires and those with logically erroneous responses, 43 of the 59 questionnaires were considered valid. The surgeons, aged 30–40 years, came from 4 different provinces in China. Among those participating in the HoLEP or TURP surgical procedures, 34 surgeons volunteered for blood pressure monitoring post-surgically. Ethical approval and written informed consent of the patients and the doctors were obtained.

All results obtained from the questionnaire were presented as means ± S.E.M. Statistically significant differences were assessed by 1-way ANOVA. All statistical analyses were performed using SPSS ver. 17.0. *P* value ≤0.05 was considered statistically significant.

## Results

**Fig. 1:**
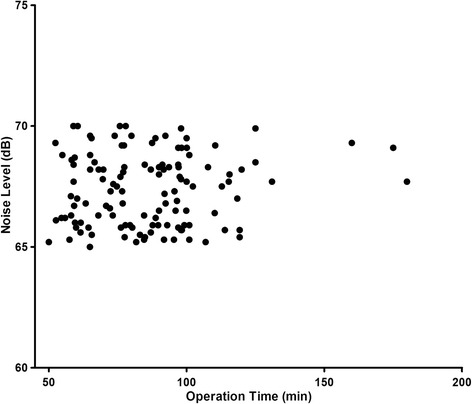
Noise levels against time since HoLEP started

As presented in Table 1, the sound level produced by the high-powered holmium laser emitter during HoLEP is greater than that produced during TURP (67.37 ± 0.13 dB VS. 46.41 ± 0.29 dB, *P* < 0.01). The sound level produced during HoLEP against time is shown in Fig. 1. The harsh noise coming from the anesthesia alarm was 64.71 ± 0.73 dB, which is close to 67 dB. The sound during TURP was 46.41 ± 0.29 dB, which is close to the baseline at 45.38 ± 0.35 dB. The noise range of 65–70 dB during HoLEP verified the safety standard set by the Occupational Safety and Health Administration (OSHA), which allows 8 h of exposure to 90 dB per day. However, 65–70 dB was considerably louder than the stated 55 dB for work requiring concentration. The range also exceeded the normal communication protective level of 60 dB.

**Table 1:** Sound level measurements

Sound source	Average sound level (dB) ± S.E.M.	Sound range (dB)	*P* value
HoLEP	67.37 ± 0.13	65.00–70.00	
TURP	46.41 ± 0.29	42.10–51.82	<0.01
Anesthesia alarm	64.71 ± 0.73	63.07–69.31	0.73
Base Line	45.38 ± 0.35	44.21–49.76	<0.01

Analysis of the responses from the surgeons indicated that in the HoLEP group, the laser emitter caused disturbance. As presented in Table 2, the score for the question “How strong was the disturbance of your communication/ concentration by noise?” was significantly higher in the HoLEP group than in the TURP group; however, no significant difference in hearing function damage was found between the 2 groups.

**Table 2:** Investigation results and analysis

Question	Average score	
	HoLEP	TURP
How strong was the auditory threshold up-regulated post-surgically?	0.8 ± 0.5	0.0 ± 0.0
How strong was your sleep disorder or dizziness?	0.6 ± 0.5	0.0 ± 0.0
How strong was the disturbance of your communication by noise?	2.2 ± 0.2^a^	0.0 ± 0.0
How strong was the disturbance of your concentration by noise?	0.8 ± 0.4^a^	0.0 ± 0.0
How strong have you felt uncomfortable after surgery because of the noise?	0.8 ± 0.5	0.0 ± 0.0

^a^ *P* < 0.05

**Table 3:** Blood pressure of surgeons

	SP (mmHg) ± S.E.M	*P* value	DP (mmHg) ± S.E.M	*P* value
HoLEP	140.9 ± 1.25		89.5 ± 0.98	
TURP	134.89 ± 1.01	<0.01	88.4 ± 1.67	0.562

*SP* systolic pressure, *DP* diastolic pressure

Analysis of results for systolic pressure (Table 3) indicated a slight increase in systolic pressure in the HoLEP group relative to that in the TURP group (140.9 ± 1.25 vs. 134.89 ± 1.01 mmHg, *P* < 0.01), whereas no significant difference was observed in the diastolic pressure results (89.5 ± 0.98 VS. 88.4 ± 1.67 mmHg, *P* = 0.562).

## Discussion

Concern for the health of physicians has drawn increasing attention because of their high-pressure working environment. Noise during surgery negatively affects surgeons. However, studies on the noise levels produced by various surgical instruments have rarely been conducted. This study aims to evaluate the sound level during HoLEP. Thus, we determined whether the sound level in the operating room during surgery was hazardous to the surgeon. Our study confirmed that the noise produced by the high-powered holmium laser emitter, which falls within the range considered safe by OSHA, does not negatively affect the surgeons’ audition in theory. Regardless, this matter should be given attention because the sound level beyond 60 dB is the upper threshold for normal communication, and 55 dB is the limit for “examinations and operations by physicians, meetings, research, and teaching.”

The safe standard range set by OSHA is designed to measure sound health in various working areas. “Table G-16” by OSHA allows 8 h of exposure to 90 dB per day beyond which hearing protection is required [8]. Meanwhile, EPA and World Health Organization deliver the standard for normal communication and work requiring concentration [9]. The sound level within the range is comfortable for doctors in the operating room and similar professionals. As suggested, the ideal degree of loudness for normal communication is 45 dB, and the maximum is 60 dB.

As is known, noise exerts negative effects on surgeons and patients. Previous studies have reported on noise in the operating theater, which exerts deleterious effects [2, 6, 10, 11]. Kurmann et al. used an intraoperative noise volume associated with subsequent surgical site infection in 35 elective open abdominal procedures [5]. In addition, the high level of noise significantly increased the incidence of postoperative complications [2]. These complications were partly attributed to the disturbance caused by the noise on the surgeon. A study found that surgeons surrounded by loud noise experienced decreased intrateam communication and interrupted conversations [2]. Another study reported that biometrically, the increased sound level enhanced both the pre- and post- operative cortisol levels and increased electrodermal potential in surgeons, which could be attributable to severe stress [12]. Thus, more effective technical and behavioral measures could be applied.

In urology, several studies suggested that the noise produced by extracorporeal shock wave lithotripsy (ESWL) can harm the hearing of the operating room personnel and the patient, although other studies contradicted this finding [13]. The difference between ESWL and the surgical procedure is that in the latter, concentration must be expended by surgeons into the surgery itself. The use of HoLEP in BHP has been increasingly prevalent because of its superior outcome and low risk of bleeding. HoLEP can potentially replace TURP as the new golden standard for BPH [1], but the noise caused by the laser emitter presents a problem. No studies have been reported on the subject; however many surgeons have complained about the upsetting side effects of the noise coming from the holmium laser emitter. To the best of our knowledge, this study is the first to report on the effect of noise produced during HoLEP on surgeons.

The results of our study showed that the baseline in the operating room was in the 45.38 ± 0.35 dB range. The sound produced during TURP, which is set as the control, was in the 46.41 ± 0.29 dB range. The noise levels produced during both procedures were considerably lower than the maximum noise for normal communication (60 dB). Although the noise of anesthesia alarm exceeded the normal communication threshold, it was transient and short-term. In the HoLEP group, the noise reached 70 dB, and the average level was about 67.37 ± 0.13 dB. The reading near the surgeon’s station verified the safety of the level of exposure set by OSHA standards, which allows 8 h of exposure to 90 dB per day. For hearing conservation, the 67.37 ± 0.13 dB level exhibited no tendency to reach the 75 dB level. Thus, no hearing hazard was observed during HoLEP. Normal communication was disturbed by the laser emitter, as determined in the study. The period lasting almost 60 min was filled with 65–70 dB noise; this degree of loudness was much higher than that by normal communication standards. During our 1-year study, 2 surgeons who performed 5 HoLEP procedures in a single day complained about tinnitus during sleep on the same day. The noise produced by the laser emitter was in the form of pulses and mainly came from the cooling system. Thus, the laser machine could be enhanced to avoid the noise.

This study has potential clinical implications. The noise level during HoLEP was within hearing conservation, but it disturbed intrateam communication and individual concentration during surgery. The noise produced by the laser emitter during HoLEP disturbed communication and concentration during surgery but did not affect hearing. In addition, post-surgical discomfort might be experienced. The major limitation of this study is its small sample size, which precludes multivariable analysis. Further studies should be conducted. Measures must also be taken to address the disturbance caused by HoLEP noise and to protect the surgeon.

## Conclusions

The noise coming from the laser emitter during HoLEP disturbs intrateam communication and the concentration of surgeons working in the operating room; however, no hearing injury is detected. Some surgeons may also experience discomfort post-surgically. Measures must be taken to resolve the disturbance caused by the noise produced during HoLEP.

## Abbreviations

BPH: Benign prostatic hyperplasia
EPA: Environmental protection agency
HoLEP: Holmium laser enucleation of the prostate
OSHA: Occupational Safety and Health Administration
TURP: Transurethral resection of the prostate
